# O082: Theories behind effectiveness: understanding behavioural change interventions to improve quality of care

**DOI:** 10.1186/2047-2994-2-S1-O82

**Published:** 2013-06-20

**Authors:** F Secci, R Edwards, W Zingg, D Pittet, A Holmes

**Affiliations:** 1 Imperial College London, London, UK; 2 University of Geneva Hospitals, Geneva, Switzerland

## Introduction

Despite increasing efforts to prevent and manage infections, hospitals struggle to reduce healthcare-associated infections (HCAI), partly because healthcare workers (HCWs) do not necessarily change their behaviour to comply with new guidelines in infection prevention and control (IPC). A number of theoretical approaches could be useful in mapping factors that influence HCW's behaviour and in supporting more effective interventions.

## Objectives

We aimed to investigate which factors influence effectiveness of interventions intended to change HCWs' behaviour in IPC, and to understand whether or not such interventions are based on theory and, if so, on which theory.

## Methods

In the context of the ECDC-funded SIGHT project, we conducted a systematic review to assess effectiveness of behaviour change and quality of care interventions, identify factors influencing effectiveness, and explore theoretical basis of interventions. We adopted an innovative tool (ICROMS) to incorporate and quality assess a broad range of methodologies.

## Results

Factors associated with guideline adherence and intervention effectiveness can be grouped into six categories: practical, individual, organisational, coercive, normative, and cultural-cognitive. Practical and individual categories including availability of facilities and attitudes, are the most frequently addressed, followed by organisational aspects, such as staff engagement. Professional norms and socialisation processes are critical, but rarely explicitly investigated. A number of studies focused on hand hygiene, but whether and how such findings are applicable to other IPC behaviours is unclear. The theoretical basis for behaviour change (e.g. Theory of Planned Behaviour and PRECEDE framework) is rarely mentioned, and focus mainly on individuals, ignoring how organisational and contextual variables may influence practice.

## Conclusion

Broadening the theoretical grounds of behaviour change interventions will allow to incorporate relevant, yet currently overlooked factors that influence HCWs' behaviour. This would facilitate planning and implementation of more successful interventions.

## Disclosure of interest

None declared.

